# Solution of Urinary Calculi

**Published:** 1842-07-16

**Authors:** 


*Solution of Urinary Calculi.*—At a recent meeting of the Medical Society of Paris, M. Petit related the following interesting case, illustrative of the action of Vichy water on urinary calculi.

The patient, a man named Jacob, had been treated during the season of 1839, by a course of Vichy waters, but had not received much benefit from them, having neglected to follow the directions given to him. On the 10th of June, 1840, he was examined by MM. Civiale, Blandin, and Berard, who frequently seized the stone between the branches of the instrument, and discovered its diameters to be thirteen, fourteen, and fifteen lines.

The man arrived at Vichy on the 21st June, and commenced his treatment on the 23d; this time he followed punctually every direction. The waters and baths were administered in the usual manner, and, in addition, the mineral waters were injected into his bladder, by means of a double syringe, two or three times a day. The dose of the waters taken by the mouth was from twelve to fifteen and often twenty glasses daily.

This treatment was borne without any inconvenience, and the other attendants at the spa took care that it was not interrupted for a single day. Towards the commencement of August the patient began to pass some fragments of calculi; several of these were lost, because he was often compelled to make water when away from home. About the 10th of September the discharge of calculous fragments ceased; the patient was now free from pain and the sensation of a foreign body, and made water freely. On the 18th he left Vichy, and on the 28th November he was examined by MM. Civiale, Blandin, and Berard, who decided that the bladder was free from stone. The patient now enjoys perfect health, and is completely free from all symptoms of catarrh of the bladder.

This is the only case in which the complete removal of the urinary calculus by the Vichy waters has been clearly demonstrated. M. Petit thinks that the calculus was destroyed rather by disintegration of its particles than by solution; he also remarks that even this case, striking as it may appear to be, leaves some doubt upon his mind, because the patient had undergone a lithotriptic sitting at the hospital Beaujon before he commenced the use of the Vichy waters. Finally M. Petit concludes that the waters of Vichy chiefly act on the mucus which unites the particles of calculi together, and in this he coincides with the opinion of M. Pelouse, which we published in our last number.—*Ibid.* April 9, 1842.

